# Age-related inflammatory biomarkers in early-onset osteoporosis in females with Gaucher disease

**DOI:** 10.3389/fendo.2025.1606218

**Published:** 2025-07-02

**Authors:** Margarita M. Ivanova, Julia Dao, Neil Kasaci, Fang Huang, Emily Nguyen, Ozlem Goker-Alpan

**Affiliations:** Translational Department, Lysosomal & Rare Disorders Research and Treatment Center, Fairfax, VA,, United States

**Keywords:** Gaucher disease, women’s health, age, osteoporosis, cytokines, inflammation

## Abstract

Gaucher disease (GD), the most common lysosomal disorder, is caused by a deficiency of the enzyme glucocerebrosidase (GCase). Accumulation of the substrate, glycosylceramide (Gb-1), and its lysosomal derivative, glucosylsphingosine, Lyso-Gb1, are associated with immune-mediated inflammation. Patients with GD experience progressive bone disease, including early-onset osteoporosis (OSR). Bone marrow infiltration with Gaucher cells and reduced bone mineral density (BMD) suggest that glycosphingolipids affect hematopoiesis, osteoclast differentiation and activity. Unlike the general population, where osteoporosis is a concern later in life, females with GD have an increased risk of BMD loss starting from adolescence, which is further impacted by pregnancy, breastfeeding, and menopause. The study’s aim was to investigate immune and inflammatory markers, focusing on early-onset osteoporosis in females. GD females and healthy controls were categorized by age: pre-menopause (<45), 45-55, and post-menopause (55+), and were further divided into three sub-cohorts: no bone complications, osteopenia (OSN), and osteoporosis (OSR). The Luminex Cytokine-96-Plex panel analysis identified 26 elevated cytokines. CD40L, APRIL, IL-35, and MIP-3β were correlated with age in healthy females but were elevated in all age categories in GD. Increased levels of Eotaxin, MCP-1, and CCL27 (CTACK) correlated with OSR. Furthermore, the age-related macrophage inflammatory protein (MIP-3β) was associated with BMD loss in female patients with GD.

**Conclusion:**

This study highlights that the ongoing release of cytokines associated with immune aging may contribute to early-onset osteoporosis in GD. By identifying age- and disease-specific cytokine signatures, including elevated levels of CD40L, APRIL, MCP-4, Eotaxin, STACK, and MIP-3β, we propose a pathophysiological link between inflammation and early-onset osteoporosis in female patients with GD.

## 1 Introduction

Gaucher disease (GD), the most common lysosomal disorder, is caused by a deficiency of glucocerebrosidase (GCase) due to *GBA1* pathogenic variants, leading to the accumulation of the substrate Gb-1 and its derivative lyso-Gb1, primarily affecting cells and tissues of the reticuloendothelial system. Lipid-laden macrophages, the hallmark of GD, have diverse phenotypic effects on disease severity and progression, including the alteration of differentiation of the mononuclear phagocyte lineage and immune dysregulation (1). Monocytes/macrophages play essential roles in bone remodeling in general. Monocytes have the ability to differentiate into osteoclasts under a suitable microenvironment and produce several osteogenic factors, which influence the activation of bone resorption and inhibit bone formation (2). Therefore, because of the alteration of mononuclear phagocyte lineage in the bone marrow due to GCase enzyme deficiency, GD patients develop a wide range of skeletal complications, including bone marrow infiltration, bone turnover failure, osteoporosis, Erlenmeyer flask deformity (EFD), cystic/lytic lesions, osteonecrosis, and osteolysis (3–6).

Early-onset low bone mineral density (BMD) with an increased risk of fractures affects women with GD at a young age (7). While osteoporosis is a later-in-life concern in women, females with GD have an increased risk of decreased BMD even during their teenage years, which further has implications for skeletal development, stability of bone density later in life, with major implications for bone loss due to pregnancy, breastfeeding, and menopause. GD is linked to immune dysregulation and chronic inflammation caused by substrate accumulation, which alters the microenvironment, promoting bone resorption while inhibiting bone formation (3, 6, 8–10). However, the underlying mechanisms for the accelerated bone density loss in females with GD remain poorly understood, and there are no standardized guidelines for the clinical management of osteopenia or osteoporosis in females across different age groups.

Aging changes in the endocrine system in females are a contributing factor to BMD loss, with increasing bone resorption and inhibition of bone formation (11). Estrogen regulates bone remodeling by targeting the nuclear factor kappa-B ligand (RANKL) in osteoblasts, promoting the expression of osteoprotegerin (OPG), and activating the Wnt/β-catenin signaling pathway (12, 13). In addition to the hormones and growth factors, cytokines such as IL-1, IL-6, and TNF-α influence bone remodeling. Inflammation has also been implicated in osteoporosis in postmenopausal women, coupled with a decrease in estrogen levels, leading to activation of bone resorption. For example, the inflammatory biomarker C-reactive protein CRP upregulates levels of cytokines such as IL-1, IL-6, IL-2, and TNF-α that positively correlated with hip and spinal bone loss in postmenopausal women (14).

Our recent findings indicate that the accumulation of Lyso-Gb-1, along with elevated levels of chitotriosidase and CCL18, are contributing factors to osteoporosis in GD. This condition is characterized by increased levels of bone turnover markers associated with bone resorption, including TRAP5b and the RANKL/OPG axis (15–17). Moreover, sclerostin, an age-related biomarker that inhibits the Wnt/β-catenin signaling pathway and suppresses bone formation, is elevated in GD (16).

Therefore, the accumulation of Gb-1 and Lyso-Gb-1 in the bone marrow triggers a cascade of downstream events, including the activation of proinflammatory cytokines that alter the functions of osteoclasts and osteoblasts in bone remodeling and lead to a loss of BMD (3, 18–20). We hypothesize that the elevation of cytokines contributes to early-onset osteoporosis in female patients with GD and could serve as biomarkers of bone pathology. The objective of this study is to define the inflammatory biomarker profiles associated with bone abnormalities in female GD patients, with the goal of identifying novel biomarkers of BMD loss and potential targets for early intervention and management. Our findings indicate that age-associated cytokines, including soluble CD40 ligand (sCD40L), a proliferation-inducing ligand (APRIL), and macrophage inflammatory protein-3β (MIP-3β), are significantly elevated in female patients with GD. Among these, MIP-3β demonstrates a strong correlation with osteoporosis. Furthermore, increased levels of Eotaxin, monocyte chemoattractant protein-1 (MCP-1), and cutaneous T-cell–attracting chemokine (CTACK) appear to contribute to bone density loss, independent of age-related changes in bone in GD.

## 2 Materials and methods

### 2.1 Subjects

All patients signed a written informed consent form before collecting and analyzing their data. Under an IRB-approved clinical protocol (Western Institutional Review Board, WIRB # 20131424), and NCT04055831, female patients with GD aged 18 to 68 years (n=30) and female healthy controls (n=22) were recruited and categorized based on age: pre-menopause (<45 years), 45–55 years, and post-menopause (55 and older) (**Table 1**). Additionally, GD females were categorized further into three groups based on BMD scores: normal BMD (N; T-score 0.03 ± 0.2; Z-scores -0.2), the osteopenia cohort (OSN; Z-score -1.07 ± 0.2, T-score -1.6), and osteoporosis cohort (OSR; Z-score -2.96 ± 0.8; T-scores-2.73 ± 0.4). Detailed medical history with an emphasis on bone disease characteristics, including prior bone surgery, bone fractures, bone pain, bone marrow infiltration, EM-flask deformity, and avascular necrosis (osteonecrosis, AVN) (**Supplementary Table 1**), was published in previous studies (16).

**Table 1:** Demographic characteristics of healthy female controls and female patients with GD.

	Healthy Control			GD		
Age	<45	45-55	>55	<45	45-55	>55
Number of patients	18	10	11	12	9	9
Minimum (Age)	19	45	56	22	45	58
Maximum (Age)	42	55	69	44	55	77
Range	23	10	13	22	10	19
Mean	32.92	48.60	61.60	33.58	50.01	64.67
Std. Deviation	7.948	3.438	4.248	7.025	3.745	6.124
Std. Error of Mean	2.294	1.087	1.343	2.028	1.248	2.041

### 2.2 Multiplex cytokine measurement

To assess the inflammatory profile, plasma samples were analyzed using the Luminex^®^ Human Cytokine/Chemokine 96-Plex Discovery Assay (Cat. #HD96; Eve Technologies Corporation, Calgary, Canada). The array features two assays (#HD48A and #HD48B) that have been conducted simultaneously from 100 µL plasma obtained from female patients with GD (n=22) and age-matched female healthy controls (n=9). These assays include premix beads for 96 cytokines from two Millipore base kits, HCYTA-60K and HCYTB-60K. Standard curves for each cytokine were prepared by serial dilution and processed in EVE Technology Corporation in parallel with samples. The kit HCYTB-60K has been used for IL-35 cytokine, labeled as mouse monoclonal anti-IL-12A, to detect the p35 subunit only, not the full heterodimeric IL-35 cytokine.

### 2.3 Enzyme-linked immunosorbent assay

Assays were performed in plasma samples as previously described, using commercial kits:sCD40L ELISA kit, (R&D System, Minneapolis, MN, USA), MCP-1ELISA(OriGene, Rockville, MD, USA), MCP-4 ELISA (MyBioSource, San Diego, CA, USA), and TNFα (Abcam, Cambridge, UK) per manufacturer’s protocols.

### 2.4 Statistical analysis

Statistical analysis was conducted using GraphPad Prism (GraphPad, San Diego, CA, USA). The study included age-matched controls for patients with GD across three groups: younger individuals (healthy controls: 32 ± 7.9 years vs. GD: 33 ± 7.0 years), middle-aged individuals (aged 45–55 years; controls: 48 ± 3.0 years vs. GD: 49 ± 3.7 years), and postmenopausal individuals (controls: 61 ± 4.2 years vs. GD: 64 ± 6.1 years) (**Table 2**).

**Table 2:** Correlation between age and cytokine levels in female healthy controls and patients with GD.

Healthy controls					
	r	R squared	P (two-tailed)	P value	Number Pars
RANTES	0.95878	0.91925	0.0006	***	7
IP-10	-0.70496	0.49697	0.0339	*	9
sCD40L	-0.68668	0.47153	0.041	*	9
APRIL	-0.76749	0.58905	0.0158	*	9
BCA-1	-0.74319	0.55233	0.0218	*	9
I-TAC	-0.69779	0.48691	0.0366	*	9
IL-33	-0.88031	0.77495	0.0017	**	9
MIP-3β	-0.7018	0.49253	0.0351	*	9
THPO	-0.72101	0.51986	0.0284	*	9
Female patients with GD					
MCP-1	0.483	0.2333	0.0196	*	23
MIG/CXCL9	0.5018	0.2518	0.0147	*	23
VEGF-A	0.455	0.207	0.0292	*	23
SCF	0.4156	0.1728	0.0486	*	23
MCP-4	0.4947	0.2447	0.0164	*	23
HMGB1	-0.4295	0.1844	0.0408	*	23
IL-16	-0.5324	0.2834	0.0089	**	23
IL-21	-0.4521	0.2044	0.0303	*	23
MIP-1δ	-0.567	0.3215	0.0091	**	20

The analysis used Pearson’s two-tailed correlation with a 95% confidence interval.

* P<0.05; **P<0.01; ***P<0.001.

Student’s t-test was used to compare biomarker levels between GD patients and controls corresponding to each age group. Two-tailed Pearson correlation coefficients were computed for cytokines at a 95% confidence interval. For group analysis, one-way analysis of variance (ANOVA) was used, followed by Kruskal–Wallis tests. A p-value of less than 0.05 indicated a statistically significant result.

## 3 Results

### 3.1 26 cytokines were significantly elevated in female patients with GD

**Figure 1:**
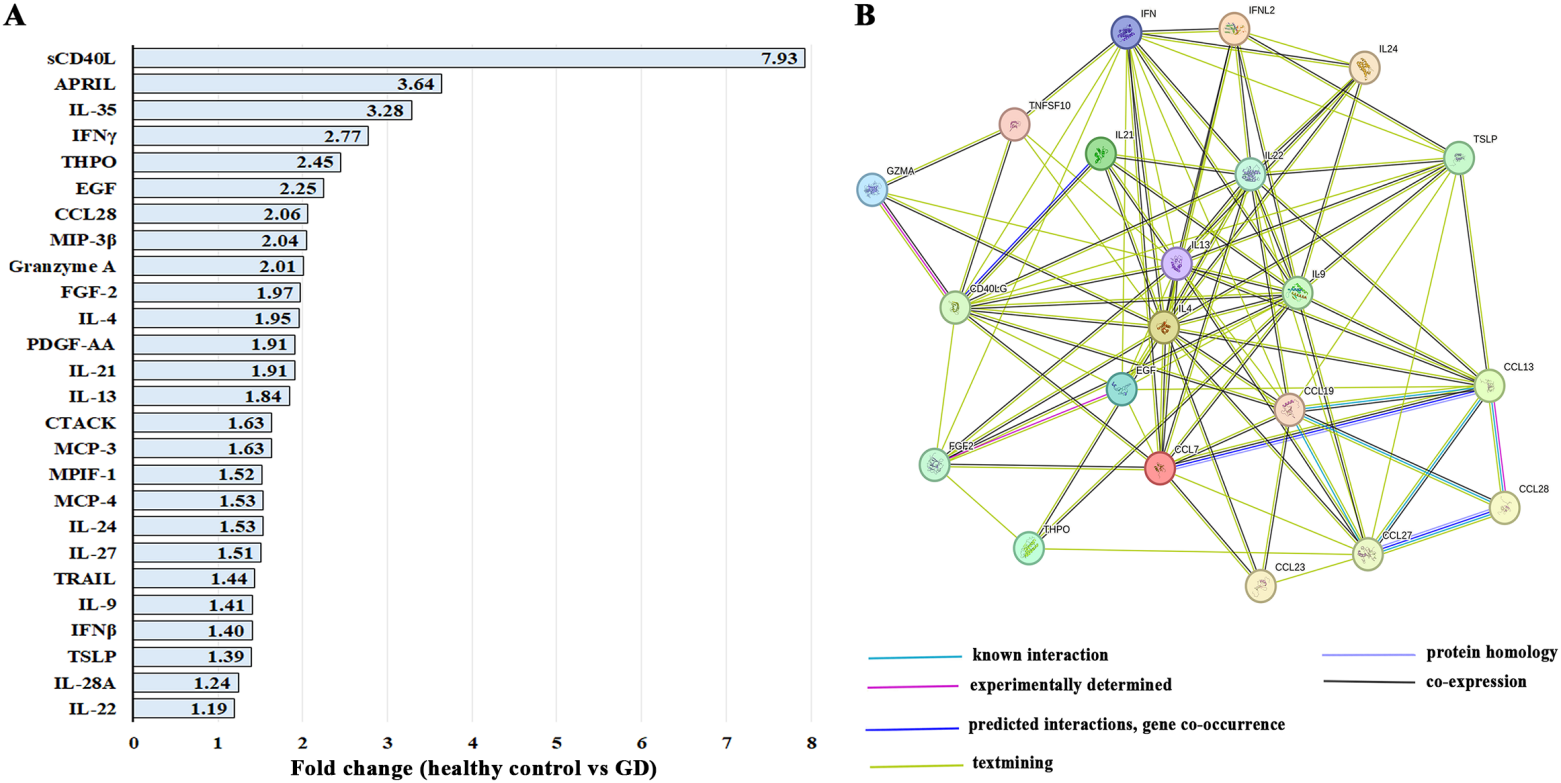
**(A)** The list of 26 significantly elevated cytokines in female patients with GD. Statistical significance was determined with a P-value of less than 0.05 using Unpaired two-tailed T-tests. **(B)** STRING interaction networks represent protein-protein associations between cytokines that are elevated in GD. Abbreviations differences: MIP-3β (CCL19), granzyme A (GZMA), CTACK (CCL27), MCP-3 (CCL7), MPIF-1 (CCL23), MCP-4 (CCL13), TRAIL (TNSF10), IL-22 (IFNL2), and IFNγ (IFN).

Gaucher disease is characterized by chronic activation of inflammatory pathways. To investigate this, circulating cytokine profiles were compared between female patients with GD and healthy female controls, regardless of age or bone pathology. In female patients with GD, 26 out of 96 cytokines were significantly elevated (**Figure 1A**, **Supplementary Table 2**). The CD40L (ligand of CD40/TNFRSF5 receptor), which is predominantly expressed in T cells, and APRIL (also known as TNFSF13), predominantly produced by myeloid cells and T cells, were the highest circulating cytokines in female patients with GD. In addition, Interleukin-35 (IL-35/p35), interferon-γ (IFN-γ), and thrombopoietin (THPO) were the most highly elevated cytokines in GD patients compared with healthy control. The increased levels of THPO may reflect compensatory hematopoietic responses to thrombocytopenia, a common hematologic manifestation in GD that results from Gaucher cell infiltration of the bone marrow (21, 22).

STRING analysis of upregulated cytokines in patients with GD revealed one large cluster with primary function in T cell activity and cytokine production, including CD40L, granzyme A (GZMA), IL-9, CCL19 (MIP-3β), CCL27 (CTACK), and CCL28 (**Figure 1B**). MCP-3 (CCL7) and MCP-4 (CCL13) are chemotactic factors that attract monocytes and eosinophils but not neutrophils (**Figure 1B**). CCL13 also attracts lymphocytes and basophils. Since only cytokines were screened using the 96-Plex Discovery Array, the biological processes and molecular functions related to cytokines were identified, including the top functions: cytokine activity, receptor binding, and ligand activity.

Interestingly, IL-35 and PDGF-AA were not represented in the STRING database, and both APRIL and IL-27 appeared as isolated cytokines without known binding interactions with cytokines that were elevated in our cohort. IL-35 is primarily involved in the activation and function of regulatory T cells. APRIL, a member of the TNF superfamily that promotes B-cell proliferation, survival, and plasma cell differentiation for antibody production, was not clustered in binding interactions with other cytokines in the GD dataset. IL-27 is known for its broad immunomodulatory functions in inflammation, autoimmunity, and cancer immunity; however, it was not clustered in binding interactions with elevated cytokines in the GD cohort (23), and its role in GD remains unclear.

### 3.2 Age-related cytokines

The phenomenon of changes in circulating cytokines as people age has been called “immune aging” (24–26). The natural aging process, environmental factors, and underlying diseases could affect immune aging. To evaluate how cytokine secretion levels changed with aging, a two-tailed Pearson correlation analysis was conducted on 96 cytokines, using a significance threshold of p<0.05.

Nine cytokines show a linear correlation with age in healthy female controls, including C-C motif chemokine ligand 5 (CCL5, or RANTES), which exhibits a positive linear correlation, and sCD40L, APRIL, IP-10, I-TAC, IL-33, MIP-3β, and THPO, which exhibit negative linear correlations (**Table 2**). In GD, a positive linear correlation between age and cytokines was verified for MCP-1, MIG/CXCL9, VEGF-A, MCP-4, and SCF, while a negative linear correlation was detected for HMGB-1, IL-16, IL-21, and MIP-1δ (**Table 2**).

### 3.3 sCD40L, APRIL, and MIP-3β plasma levels correlated with age in healthy controls but remain elevated in GD patients

**Figure 2:**
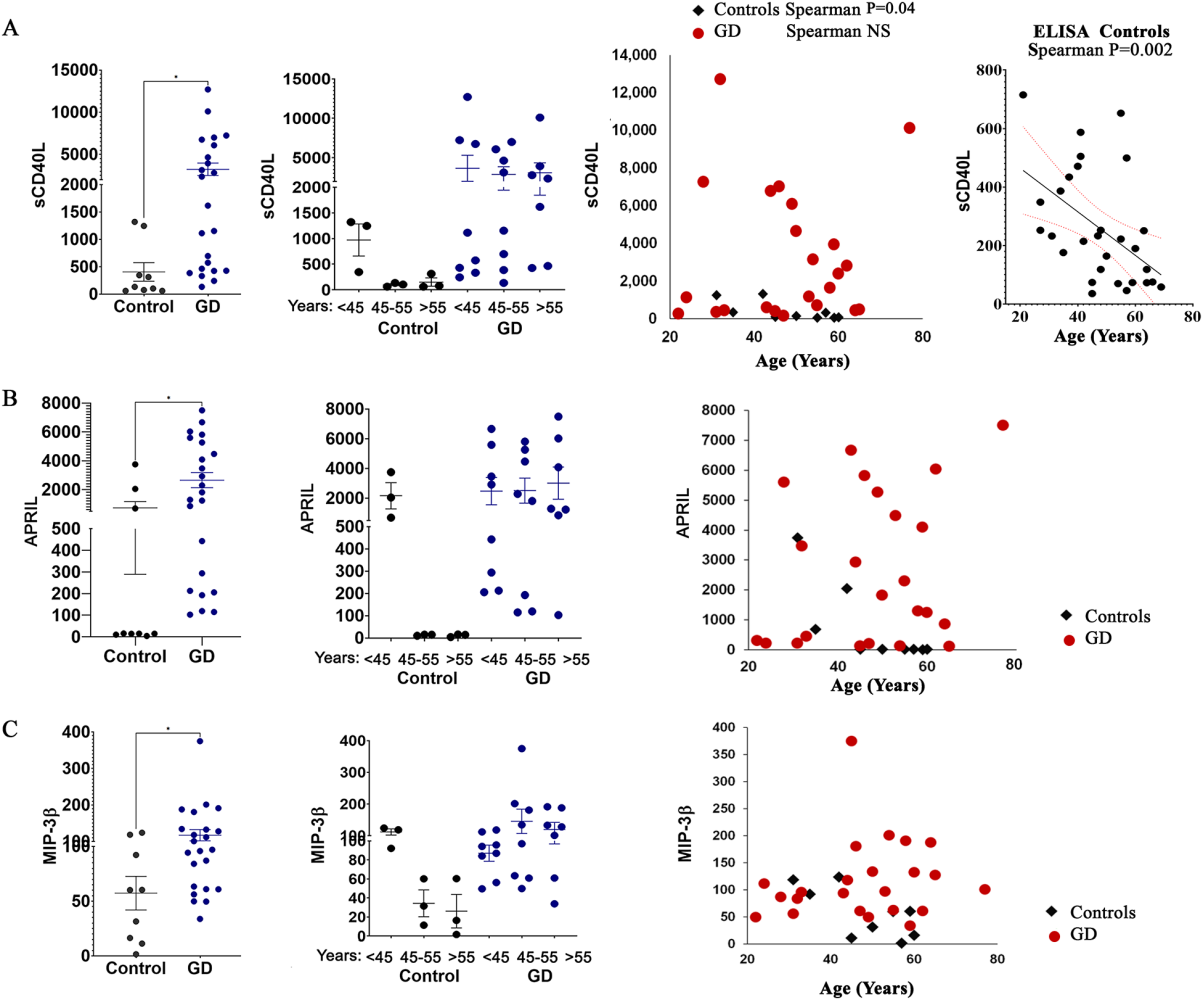
The plasma levels of sCD40L **(A)**, APRIL **(B)**, and MIP-3β **(C)** in female patients with GD compared to healthy controls. Statistical significance was determined using Unpaired two-tailed t-tests with a significance level of P < 0.05. Additionally, the cytokine levels of sCD40L, APRIL, and MIP-3β were analyzed in relation to age. * P value <0.05.

sCD40L plays a significant role in immune aging, as its expression on T cells decreases with age, leading to impaired B cell activation and reduced interactions between T and B cells (27). As a result, a decline in sCD40L expression is associated with a weakened immune response. In our study, sCD40L levels decreased in the healthy female control group aged 45–55 years and those 55 years and older (**Figure 2A**). In patients with GD, the level of sCD40L tends to be significantly elevated, and this occurs regardless of age (**Figure 2A**). Pearson correlation analysis of the Multiplex 96-plex discovery array demonstrates that healthy controls show a significant negative linear correlation between age and circulating sCD40L. Furthermore, screening 30 healthy controls using sCD40L ELISA confirmed a decrease in plasma sCD40L levels with age (**Figure 2A**).

APRIL, which is expressed by monocytes, macrophages, neutrophils, dendritic cells, T-cells, and osteoclasts, is released into the circulation in a soluble, active form after cleavage (28). As a ligand, APRIL interacts with heparan sulfate proteoglycans, the transmembrane activator and calcium modulator cyclophilin ligand interactor (TACI), and B cell maturation antigen (BCMA) (29). APRIL has been associated with various autoimmune diseases, including systemic lupus erythematosus, rheumatoid arthritis, diabetes, and activation of bone resorption in multiple myeloma (30). Moreover, circulated levels of APRIL are inversely correlated with age in healthy subjects (31). Our data, consistent with previous publications, show that the secretion level of APRIL is much higher in young subjects and decreases with age in females (**Figure 2B**). However, in female patients with GD, the level of APRIL is higher regardless of age (**Figure 2B**).

Similar patterns have been observed for macrophage inflammatory protein-3β (MIP-3β or CCL19). The circulating level of MIP-3β correlates with age in healthy females but is elevated in patients with GD regardless of age (**Figure 2C**). Plasma MIP-1β, like MIP-1α and MIP-4 (CCL18) were elevated in patients with GD (32, 33). To our knowledge, this is the first evidence showing an increase in the levels of MIP-3β in patients with GD.

### 3.4 The levels of MCP-1, MCP-4, IL-21, and IL-16 correlate with age

**Figure 3:**
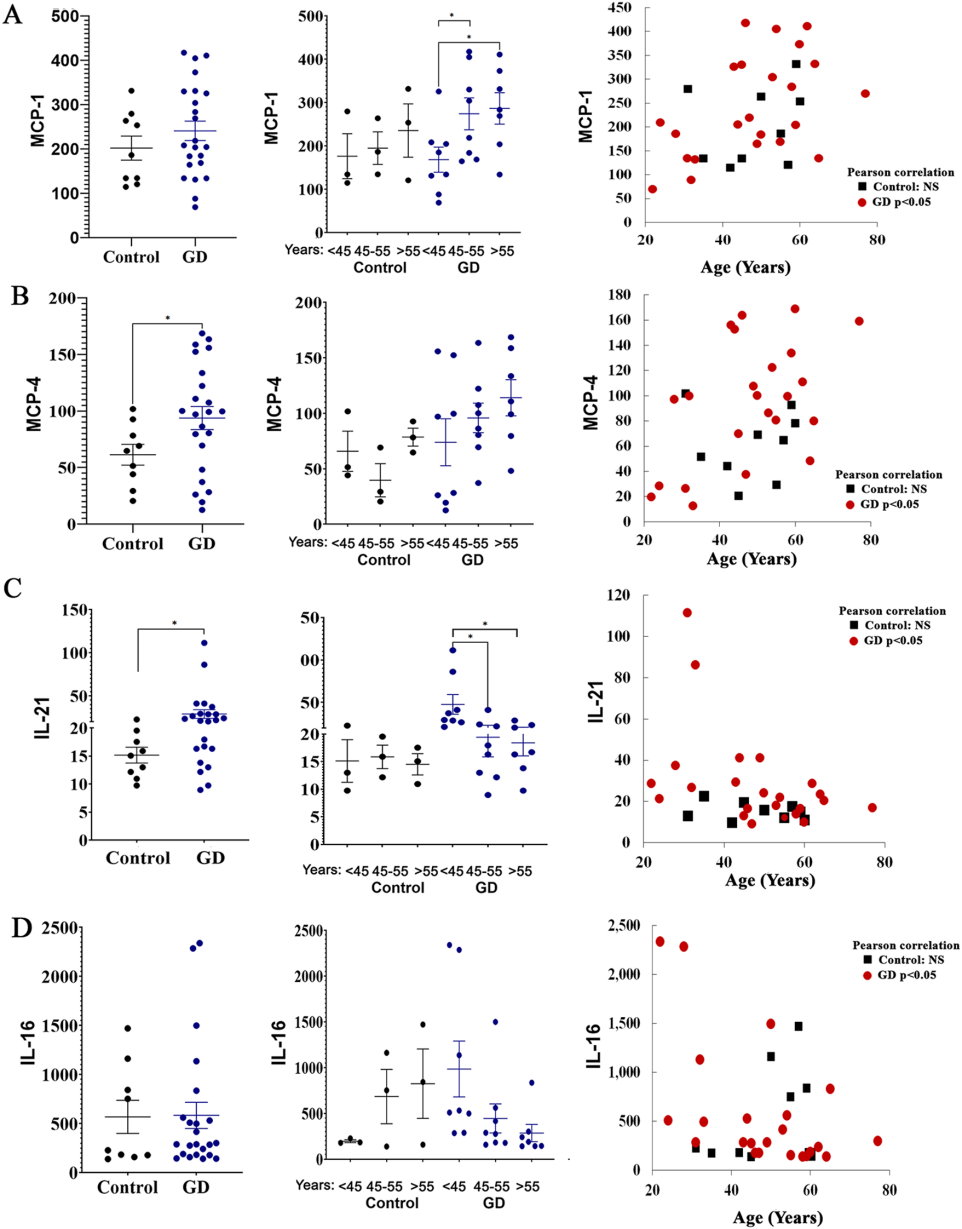
The plasma levels of MCP-1 **(A)**, MCP-4 **(B)**, IL-21 **(C)**, and IL-16 **(D)** in female patients with GD compared to healthy controls. Statistical significance was determined using Unpaired two-tailed t-tests with a significance level of P < 0.05. Additionally, the cytokine levels of MCP-1, MCP-4, IL-21, and IL-16 were analyzed in relation to age. Pearson linear correlation analysis showed significant differences with a p-value <0.05. * P value <0.05.

MCP-1 (CCL2) and MCP-4 (CCL13) are chemokines that play a role in inflammation, primarily attracting monocytes and T cells. MCP-4 also attracts eosinophils and basophils to manage inflammatory responses to allergens. MCP-1 is elevated in GD and contributes to inflammation in GD due to macrophage activation (1). However, the role of MCP-4 in GD is unknown. Multiplex cytokine panel analysis indicated no differences in MCP-1 levels between healthy females and patients with GD (mean levels in GD, 241+/-21 and healthy controls, 202+/-27) (**Figure 3A**). Due to overlapping standard errors, the means have not reached significance. Therefore, we compared MCP-1 levels in 22 healthy controls and 30 female patients with GD using the MCP-1 ELISA to validate the above data. The inclusion of a larger cohort of subjects and healthy controls confirmed the elevation of the circulating levels of MCP-1 among females with GD compared to their healthy counterparts (**Supplementary Figure 1**) Slight differences between the multiplex assay and ELISA observed were likely due to a limited number of healthy controls included in the multiplex analysis. MCP-1 levels increased in perimenopausal and postmenopausal females (**Figure 3B, C**). Multiple studies confirm that MCP-1 levels increase with age in patients with osteoporosis, osteoarthritis, and rheumatoid arthritis, contributing to inflammaging (14, 34). The majority of females with GD showed elevated levels of MCP-4, a finding not previously reported (**Figure 3A**). Moreover, the elevation of the MCP-4 level was increased with age in GD female patients (**Figures 3B, C**). The lack of age-specific data for MCP-4 in healthy controls suggests it’s not as commonly studied as MCP-1 in this context. Measurement of MCP-4 in 34 healthy female controls showed no correlation between age and MCP-4 levels (**Supplementary Figure 1**), therefore, aging MCP-4 levels are associated with GD.

IL-21, produced by CD4+ T cells, plays a crucial role in modulating dendritic cells and macrophages (35). Furthermore, a recent study also showed a high level of IL-21 in GD (36). All female patients with GD aged 45 years and younger showed increased IL-21 levels (**Figure 3D**). In patients with GD who are 45 years old and older, the levels of IL-21 are lower compared to younger patients (**Figure 3C**). Further investigation is needed to interpret results related to age and secretion of IL-21 levels in GD.

Elevated IL-16 is associated with obesity-related inflammatory responses (37, 38). In our cohort, increased IL-16 levels were observed in healthy female controls who are 45 and older. In contrast, female patients with GD exhibited an age-associated decline in IL-16 plasma levels. Interestingly, among GD patients, three of the four individuals with the highest IL-16 were classified as overweight, with body mass index (BMI) values ranging from 28 to 33. These findings suggest a potential influence of adiposity on IL-16 expression in the context of GD; however, the sample size is small to draw a conclusion.

### 3.5 Age-dependent MIP-3β elevations in GD female patients correlate with osteoporosis

**Figure 4:**
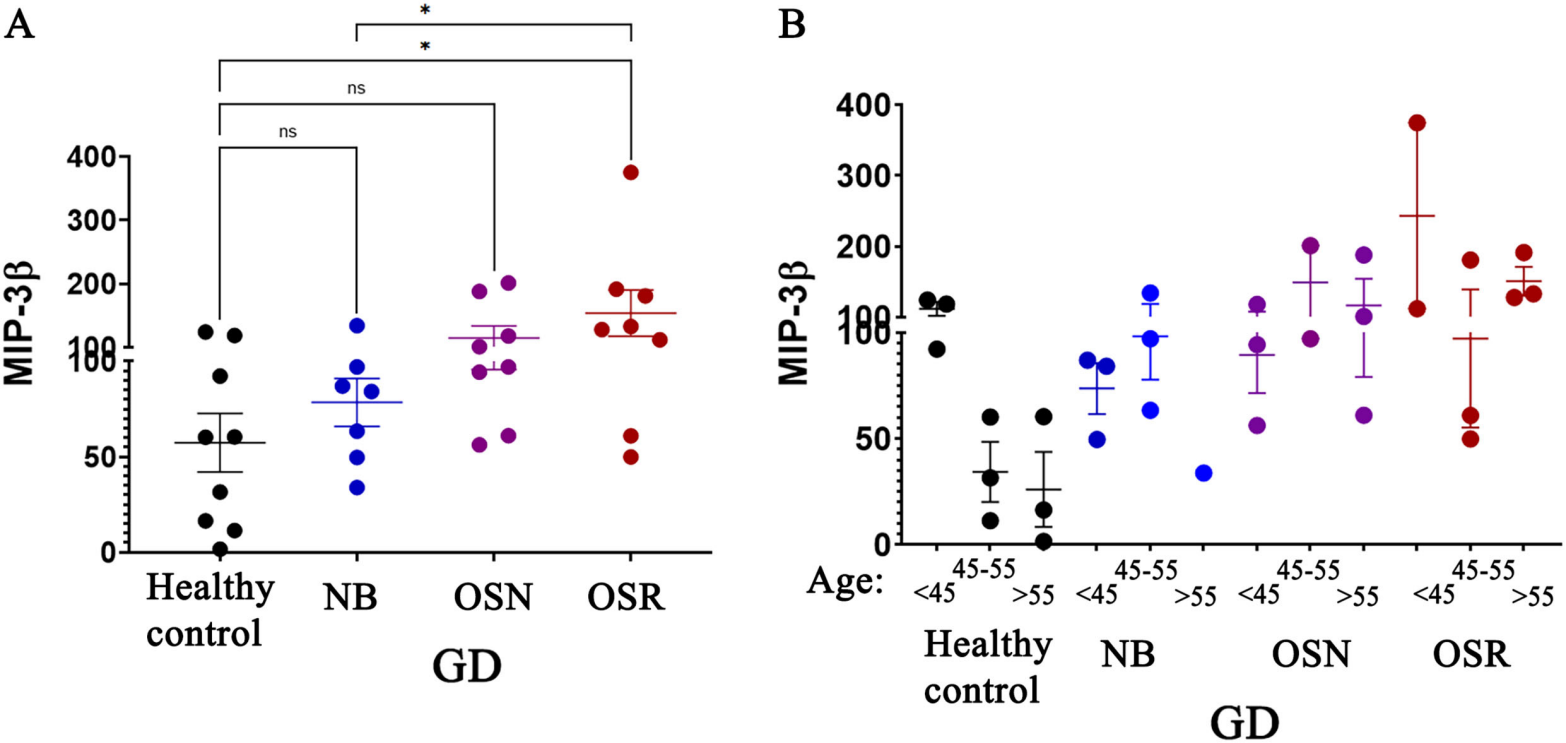
Age-dependent macrophage inflammatory protein (MIP-3β) correlates with BMD loss in female patients with GD. Plasma MIP-3β Levels: **(A)** The levels of MIP-3β in healthy control subjects and in patients with GD who have no bone complications (N), osteopenia (OSN), and osteoporosis (OSR). Statistical significance was determined with P < 0.05, using ANOVA and T-tests. **(B)** The MIP-3β levels in GD patients were analyzed in relation to age. The cohort was divided into three groups based on age: under 45 years, 45–55 years, and over 55 years. These groups were further categorized based on osteopenia (OSN) and osteoporosis (OSR) diagnosis. * P value <0.05, NS - non-specific.

Analysis of age-dependent cytokines in healthy controls, CD40L, APRIL, and MIP-3β, that were elevated in GD, was assessed for potential association with osteopenia and osteoporosis. The results showed that MIP-3β levels were elevated in female patients with osteoporosis compared to healthy controls and GD patients with normal BMD (NB) (**Figure 4A**). When cohorts were stratified by age group, results verified that GD females aged 45 and older had elevated levels of MIP-3β. This difference is more pronounced in females with osteopenia and osteoporosis. Although MIP-3β is not identified as a mediator of osteoporosis, it may contribute indirectly by amplifying inflammatory environments that affect bone remodeling, highlighting the potential role as a biomarker in bone pathology. However, a more detailed investigation is necessary to establish the role of MIP-3β in GD.

In contrast to MIP-3β, CD40L and APRIL showed no association with decreased BMD, as their levels did not show significant differences between patients with osteoporosis and healthy controls. This indicates that CD40L and APRIL are unlikely to play a significant role in BMD reduction.

### 3.6 Non-age-dependent cytokines correlate with osteoporosis in female patients with GD

**Figure 5:**
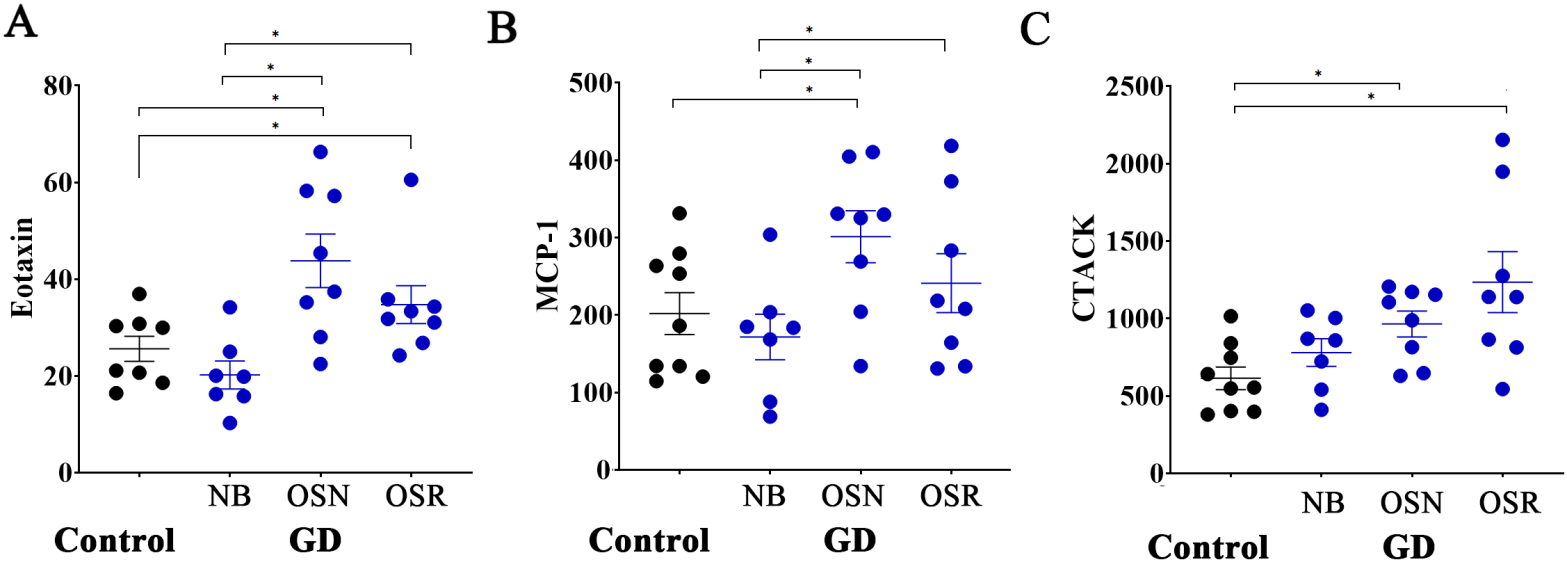
Age-independent Eotaxin, MCP-1, and CTACK correlate with osteoporosis in female patients with GD. **(A)** The levels of Eotaxin in healthy control subjects (Control) and in patients with GD who have no bone complications (N), osteopenia (OSN), and osteoporosis (OSR). **(B)** The levels of MCP-1 in healthy controls and GD patients without bone complications (N), osteopenia (OSN), and osteoporosis (OSR). **(C)** The levels of CTACK in healthy controls and GD patients without bone complications (N), osteopenia (OSN), and osteoporosis (OSR). Statistical significance was determined with ^*^P < 0.05, two-tailed T-tests.

Next, we focused on cytokines that do not vary with age but may be involved in the loss of BMD independently of the natural aging changes in the bones. Three biomarkers, Eotaxin, MCP-1, and CCL27 (CTACK), were associated with reduced BMD in female patients. Eotaxin (CCL11) is a chemokine involved in inflammatory responses. Recent publications indicate that Eotaxin may contribute to bone loss by increasing bone resorption by binding to the CCR3 receptor, which is present in osteoclasts (39, 40). In our study, Eotaxin levels were higher in patients with osteopenia and osteoporosis, with no significant differences between the two conditions, suggesting that Eotaxin might be involved in the initial phases of reduction in bone density and could be a promising option for the early detection of BMD loss (**Figure 5A**). MCP-1 (CCL2) is an inflammatory chemokine that attracts monocytes and macrophages. Elevated MCP-1 boosts the differentiation and migration of precursor cells into mature osteoclasts, leading to enhanced bone resorption (41–43). Therefore, numerous studies demonstrate that individuals with osteoporosis show higher MCP-1 levels. In GD, the accumulation of Gb-1 and lyso-Gb-1 in macrophages triggers the secretion of MCP-1 (1). The chronic inflammatory environment due to elevated MCP-1 levels contributes to splenomegaly, hepatomegaly, and bone pathology. High levels of MCP-1 are associated with GD, but treatments such as ERT effectively lower these levels by reducing glucocerebroside through the delivery of the deficient enzyme (44). Therefore, as we mentioned before, we did not observe considerably elevated levels in GD patients (**Figure 3A**, **Supplementary Figure 1**); however, elevated levels of MCP-1 were correlated with OSN and OSR in female patients (**Figure 5**, **Supplementary Figure 1**).

Cutaneous T-cell-attracting chemokine (CCL27 or CTACK) is involved in immune cell trafficking, skin homeostasis, and inflammatory responses. However, its potential role in osteoporosis or GD remains unclear and has not been investigated as comprehensively as Eotaxin or MCP-1. Here, we did not observe elevated levels of CCL27 in all patients with GD when compared to healthy controls. However, elevated levels of CCL27 were correlated with OSN and OSR in female patients compared to healthy controls (**Figure 5B**). In summary, a more comprehensive analysis of CCL27 is necessary, with an increased sample size for further validation. Linear correlation analysis of the Z-scores and T-scores of BMD and Eotaxin, MCP-1, and CTACK showed no significant differences in cytokine levels and BMD (**Supplementary Tables 3**, **4**), likely due to the limited statistical power caused by a small sample size after division into BMD scores.

Therefore, Eotaxin, MCP-1, and CTACK may contribute to bone density loss and impact it beyond age-related changes in bone. To further investigate the potential associations of biomarkers with Z or T-scores, increasing the number of cohorts in future studies is necessary to improve the data.

## 4 Discussion

Chronic inflammation is a well-established contributor to the pathophysiology of GD, including in relation to bone complications. However, a critical gap remains in our understanding of early-onset osteoporosis in females with GD and its association with age-related cytokines. This manuscript presents findings from a noninterventional, observational study examining the relationship between circulating inflammatory cytokines, age- and sex-specific bone turnover markers, and structural bone changes. The study focuses on BMD loss across distinct female age groups: premenopausal (<45 years), perimenopausal (45–55 years), and postmenopausal (>55 years) women.

The structural bone changes are amplified due to hormonal factors, particularly after menopause, as estrogen levels decline. Estrogen typically has a protective effect on bone density by inhibiting osteoclast activity, so its reduction may intensify inflammation-driven bone loss in GD. The risk of developing osteoporosis is approximately 14% in women between 50–59 years old in general; the projected risk of osteoporosis in women with GD is up to 30% at premenopausal ages (45–47). For postmenopausal women, the risk is higher due to the effect of estrogen decline. However, exact figures for postmenopausal females with GD alone are not consistently presented in the publications related to registries (46). Low BMD could be observed during childhood in GD and is most pronounced during the peak bone mineralization that occurs in the second and third decades of life (47). Therefore, Early-onset osteoporosis is common in women with GD due to loss of BMD at a young age.

The accumulation of Gb-1 and its deacylated form, Lyso-Gb-1, affected bone metabolism in GD in two ways: directly affecting bone cells and inflammation-driven bone loss (3, 15, 18–20, 48, 49) Research, including studies in mouse models, has shown that these sphingolipids accumulate in osteoblasts, which are bone-forming cells, inhibiting pathways like protein kinase C, which are essential for bone formation (19). Osteoblasts derived from GD iPSCs showed lower expression of differentiation markers, and the deposition of bone matrix proteins and minerals was impaired, alongside reduced canonical Wnt/β-catenin signaling (50). Our recent findings have shown that the elevation of inhibitors of the Wnt/β-catenin pathway correlates with bone pain, Erlenmeyer flask deformity, and bone marrow infiltration in patients with GD (16).

Inflammation-driven BMD loss significantly contributes to osteopenia and osteoporosis (51, 52). Under normal conditions, bone remodeling maintains healthy bone density, but inflammation disrupts the delicate balance between bone resorption and formation, leading to the most common bone pathology in aging females – osteoporosis (52, 53). Concurrently, the role of genetic factors on early-age osteoporosis highlights a more significant genetic impact on BMD pathology. GD is a rare genetic disease where mutations of the *GBA1* gene link to enzyme deficiency and substrates accumulation of Gb-1 and lyso-Gb-1 in various tissues, including the bone marrow. This accumulation triggers chronic inflammation, which also disrupts normal bone remodeling processes. Therefore, mutation of the *GBA1* gene may be one of the genetic factors in the early onset of osteoporosis. Our recent findings have shown that GD patients with osteopenia and osteoporosis have elevated levels of bone biomarkers related to the activation of bone resorption and dysregulation of the RANKL-OPG-RANK and Wnt/β-catenin pathway (15–17). One of these biomarkers, sclerostin, is age-related in healthy females and associated with bone pathology in GD (16). However, in females with GD, the elevation of sclerostin started early and further increased with age. These observations showed that structural bone changes that occur during the course of GD accelerate the aging bone loss in women.

Pro-resorptive and anti-resorptive cytokines influence bone homeostasis (53, 54). Inflammatory cytokines (e.g., IL-1, IL-6, TNF-α, IFN-γ) ramp up osteoclast activity (55), which breaks down bone while simultaneously hindering osteoblasts, the bone-builders. MCP-1 recruits osteoclast precursors in inflammatory bone diseases such as rheumatoid arthritis (56), and the elevation of MCP-1 may be a novel predictive marker for the detection of early bone loss in animal models (57, 58). Eotaxin promotes the migration of pre-osteoclasts, which then differentiate into mature osteoclasts, enhancing bone resorption and potentially serving as a candidate biomarker for postmenopausal osteoporosis (39).

TNF-α upregulates RANKL expression on synovial fibroblasts and stromal cells, activating osteoclast differentiation and contributing to bone resorption, as observed in inflammatory conditions, for example, rheumatoid arthritis (59–61). In GD, Gaucher cell infiltration in bone marrow further amplifies local inflammation, elevating TNF-α and other cytokines (e.g., IL-1, IL-6), which disrupt the bone microenvironment, impair hematopoiesis, and bone remodeling. Elevated levels of TNF-α in GD vary widely, from normal to 2.5 times the highest, with higher levels in severe neuronopathic forms (17, 62, 63). A pilot study of 17 type 1 GD patients found a correlation between TNF-α levels and the -308 G→A promoter polymorphism, with homozygous patients (A/A) exhibiting lower TNF-α levels and milder disease phenotypes compared to heterozygotes (G/A), suggesting genetic variability influences disease severity (64). In this study, TNF-α levels were found to be higher in female patients compared to healthy controls; however, this difference was not statistically significant when measured using the MultiPlex assay. In contrast, the ELISA assay revealed a significant increase in TNF-α levels (**Supplementary Figure 2**). Furthermore, the elevated TNF-α levels in female patients did not correlate with a decrease in BMD (**Supplementary Figure 2**).

IFN-γ can stimulate osteoclast formation and bone loss *in vivo*, particularly in models of postmenopausal osteoporosis. Our study shows chronic inflammation in female patients, with significant elevations in cytokines that play major roles in T cell activity and cytokine production, including sCD40L, APRIL, IL-35, IFN-γ, and THPO. CD40L binds to CD40, is involved in bone metabolism, and leads to an increased risk of osteoporosis in women (65). According to the literature, no studies demonstrate that sCD40L is significantly elevated in patients with GD. But, connections may exist over the macrophage’s activation and inflammation, but these are speculative. Further investigation would be needed to clarify any potential link. APRIL promotes B cell survival and antibody production; however, its overexpression can lead to excessive B cell activity, which contributes to autoimmune diseases. IL-35’s primary role is in regulating T-cell responses. To our knowledge, APRIL or IL-35 cytokines are novel biomarkers in GD research.

Aging is associated with a dynamic shift in circulating cytokine profiles. These changes reflect an imbalance between pro-inflammatory and anti-inflammatory signals, which usually leads to the upregulation of pro-inflammatory cytokines and a concomitant decline in anti-inflammatory signals (24–26). This can be accelerated in females due to hormonal shifts (menopause age), where declining estrogen levels remove an inhibitory control on osteoclast activity, amplifying the inflammatory damage. This effect is further intensified in the context of GD, where chronic inflammation is perpetuated by the accumulation of Gb-1 and its bioactive metabolite Lyso-Gb-1. Inflammation promotes bone loss, contributing to skeletal fragility and compounding disease-associated morbidity. Age-stratified analysis revealed that the level of 9 cytokines changes due to hormonal shifts in healthy females, including RANTES, IP-10, sCD40L, APRIL, BCA-1, I-TAC, IL-33, MIP-3β, and THPO. Some of these data are consistent with previous studies that aging is associated with an increased level of RANTES (66), and a decline in IL-33 with advancing age (67, 68).

A correlation analysis between age and cytokine levels revealed that elevated levels of plasma MCP-1, MIG/CXCL9, SCF, and MCP-4 are positively associated with age in GD patients but not in healthy controls. Moreover, MCP-4 levels were significantly elevated in GD compared to healthy controls and continue to increase with advancing age. MCP-1, MIG/CXCL9, and SCF are key regulators of macrophage behavior in GD, which is central to GD pathogenesis (1, 69, 70). If MCP-1 recruits monocytes that differentiate into lipid-laden Gaucher cells, SCF supports macrophage development and survival. MIG/CXCL9 modulates the inflammatory environment via T-cell recruitment and directly affects macrophage activation. Together, these cytokines contribute to macrophage accumulation, activation, and dysfunction, which are hallmarks of GD pathology (1, 70).

Although these biomarkers are typically elevated in treatment-naïve GD patients, the majority of individuals in our cohort were on ERT or SRT. Therefore, an elevation in MCP-1 levels was not anticipated. While multiplex data suggested that MCP-1 levels were not significantly increased in GD, an ELISA assay with a larger sample size confirmed that approximately 45% of patients have elevated MCP-1 levels. Moreover, females with elevated MCP-1 levels are 45 years or older and have BMD loss. Unlike MCP-1, MCP-4 (CCL13) has not been studied in the context of inflammation and macrophage regulation in GD. This chemokine represents inflammation and immune responses and holds potential as a biomarker for rheumatic diseases, skin conditions, or cancer (71). Our findings demonstrated a significant elevation of plasma MCP-4 in women with GD compared to healthy controls. Furthermore, MCP-4 levels appear to increase progressively with age, suggesting its potential efficacy as a biomarker of inflammation-associated bone pathology in aging females with GD.

Chemokine MIP-3β (CCL19) is involved in immune responses, particularly in attracting immune cells to areas of inflammation. Fibroblastic reticular cells (FRCs) generate the three-dimensional structure of lymph nodes and coordinate estrogen-dependent immune responses by expressing the chemokine MIP-3β, which attracts immune cells (72). Moreover, estrogen signaling in MIP-3β-expressing bone marrow stromal cells is essential for maintaining bone health. Decreasing estrogen signaling in these cells leads to trabecular bone loss due to increased bone resorption observed in a mouse study (73). Our data indicates that lower estrogen levels in healthy females may correlate with decreased secretion of MIP-3β, findings that is also supported by publications (74). Moreover, MIP-3β may be a potential biomarker for BMD loss associated with aging in GD.

Two other cytokines, Eotaxin and CCL27 (CTACK), were associated with an age-independent decrease in BMD in female patients with GD. Eotaxin may be a novel biomarker for osteoporosis, given its significant role in osteoclast migration and its ability to enhance osteoclastic bone resorption under inflammatory conditions (39, 75). Conversely, the potential role of CTACK in bone pathology in GD remains unclear except in the context of myeloma, which may play a role in the bone marrow microenvironment (76). From our data, CTACK emerges as an osteoporosis biomarker.

Enzyme replacement therapy (ERT) and substrate reduction therapy (SRT), the standard treatments in GD, can reduce inflammation and improve bone health to some extent, albeit more slowly over time, but they don’t fully reverse skeletal damage (16, 46, 77). Numerous clinical studies have demonstrated that SRT (eliglustat) and ERT exhibit comparable efficacy in reducing biomarkers, including inflammatory biomarker CCL18, and enhancing visceral outcomes (77, 78). Bone marrow infiltration, avascular osteonecrosis improved with eliglustat, along with a reduction in osteoclastogenic biomarkers such as RANKL and osteopontin; however, BMD showed minimal improvement, suggesting a slower skeletal response (78–81). This suggests that targeting inflammation directly, alongside with ERT or SRT therapies, could be key to managing osteoporosis in these patients.

The chronic inflammation in GD alters the normal age-related patterns of CD40L, APRIL, and MIP-3β, resulting in their sustained elevation with age. This disruption may interfere with bone turnover and accelerate BMD loss, contributing to the development of osteoporosis. Among these cytokines, MIP-3β stands out for its robust association with BMD loss in female GD patients. MIP-3β significantly higher levels in GD patients with osteoporosis suggest a direct role in bone resorption and support its utility as a potential biomarker for tracking disease-related bone density loss. Unlike classical postmenopausal osteoporosis, which arises from estrogen deficiency and manifests later in life, GD-associated osteoporosis frequently emerges in premenopausal females. This early onset reflects the unique impact of chronic inflammation, Gb-1/Lyso-Gb-1 accumulation, and macrophage activation on bone homeostasis. Moreover, the chronic inflammation and immune dysregulation in GD accelerate the breakdown of trabecular and cortical bone architecture, heightening the risk of fractures and functional impairment at a young age.

In summary, this study highlights the critical role of inflammation in the development of early-onset osteoporosis in females with GD. By identifying age- and disease-specific cytokine signatures, including the elevation of CD40L, APRIL, MCP-4, Eotaxin, STACK, and MIP-3β, we established a pathophysiological axis linking inflammation to early-age osteoporosis in GD. These findings not only expand our understanding of mechanisms of bone remodeling in GD but also point toward novel biomarkers and therapeutic targets that may improve the management of bone health in females with GD.

## Data Availability

The original contributions presented in the study are included in the article/**Supplementary Material**. Further inquiries can be directed to the corresponding author.

## Supplementary material

Supplementary Figure 1 Circulated levels of MCP-1, MCP4 and TNF-α. **(A)** The levels of MCP-1 in healthy control subjects (Control) and patients with GD. **(B)** The levels of MCP-1 in Control and in patients with GD categorized by bone status: no bone complications (N), osteopenia (OSN), and osteoporosis (OSR). Statistical significance was determined with ^*^P < 0.05, two-tailed T-tests. **(C)** The correlation between age and the circulating MCP-4 level in healthy controls. The MCP-4 level was measured using ELISA. **(D)** The levels of TNF-α in healthy control subjects and patients with GD. The TNF-α level was measured using ELISA. Statistical significance was determined with *P < 0.05, two-tailed T-tests. **(E)** TNF-α levels in female patients with GD categorized by bone status: no bone complications (N), osteopenia (OSN), and osteoporosis (OSR).
