# Supplementary material for: Age-related inflammatory biomarkers in early-onset osteoporosis in females with Gaucher disease
Source: Front Endocrinol (Lausanne). 2025 Jul 2;16:1606218. doi: 10.3389/fendo.2025.1606218 (PMC12263375; doi:10.3389/fendo.2025.1606218)
Supplement: Supplementary Figure 1 — Circulated levels of MCP-1, MCP4 and TNF-α. (A) The levels of MCP-1 in healthy control subjects (Control) and patients with GD. (B) The levels of MCP-1 in Control and in patients with GD categorized by bone status: no bone complications (N), osteopenia (OSN), and osteoporosis (OSR). Statistical significance was determined with *P < 0.05, two-tailed T-tests. (C) The correlation between age and the circulating MCP-4 level in healthy controls. The MCP-4 level was measured using ELISA. (D) The levels of TNF-α in healthy control subjects and patients with GD. The TNF-α level was measured using ELISA. Statistical significance was determined with *P < 0.05, two-tailed T-tests. (E) TNF-α levels in female patients with GD categorized by bone status: no bone complications (N), osteopenia (OSN), and osteoporosis (OSR). [file DataSheet1.docx]

Supplementary Material

# Supplementary Tables 1

An individual's medical history includes age, type of therapies (enzyme replacement therapy, ERT, and substrate reduction therapy, SRT), GBA1 sequencing variants, splenectomy, bone pain, and characteristics of bone medical history, including Z- and T-scores. Samples that were analyzed using Multiplex assays were highlighted.

|  | **Multiplex Assay** | **Therapy** | **Age** | **Sequence** | **Splenectomy** | **Bone surgery** | **Bone pain** | **Bone marrow infiltration** | **EM-flask deformity** | **Cystic** | **Pathological fractures** | **AVN** | **Z score** | **T**  **score** |
| --- | --- | --- | --- | --- | --- | --- | --- | --- | --- | --- | --- | --- | --- | --- |
| **Age group <45** | | | | | | | | | | | | | | |
| Normal bone | Yes | ERT 5-10 | 22 | N370S/F213I | NO | NO | YES | YES | YES | NO | NO | NO | -0.2 |  |
|  | Yes | SRT | 32 | N370S/N370S | NO | NO | NO | NO | NO | NO | NO | NO | -0.8 |  |
|  | Yes | ERT >10 | 28 | L444P/L444P | NO | NO | NO | NO | NO | NO | NO | NO | -0.3 |  |
|  |  | ERT >10 | 41 | N370S/W381X | NO | NO | YES | YES | NO | NO | NO | YES | -0.6 |  |
|  |  | ERT 5-10 | 38 | N370S/L444P | NO | NO | NO | NO | NO | NO | NO | NO | -0.5 |  |
| Osteopenia |  | ERT 1-5 | 32 | N370S/N370S | NO | YES | NO | YES | YES | NO | NO | NO | -1.30 |  |
|  |  | ERT 5-10 | 35 | L444P/N370S | NO | NO | NO | NO | NO | NO | NO | NO | -1.10 |  |
|  | Yes | SRT | 43 | N370S/R463C | NO | NO | NO | YES | NO | NO | NO | NO | -1.10 |  |
|  | Yes | ERT >10 | 31 | N370S/N370S | NO | NO | YES | NO | NO | NO | NO | NO | -1.80 |  |
|  |  | ERT >10 | 44 | N370S/R120Q | NO | NO | YES | YES | NO | NO | NO | NO | -1.70 |  |
| Osteoporosis | Yes | ERT 1-5 | 24 | N370S/L444P | NO | NO | YES | NO | NO | NO | NO | NO | -3.2 |  |
|  | Yes | ERT >10 | 33 | N370S/N370S | NO | NO | NO | YES | NO | NO | NO | NO | -2.8 |  |
| **Age group 45-55** | | | | | | | | | | | | | | |
| Normal bone | Yes | ERT >10 | 50 | N370S/L444P | NO | YES | YES | YES | NO | YES | NO | YES | -0.4 |  |
|  | Yes | SRT | 55 | N370S/R463C | NO | NO | NO | YES | YES | NO | NO | YES | -0.4 |  |
|  | Yes | ERT 5-10 | 53 | N370S/N370S | NO | NO | NO | YES | YES | NO | NO | NO | -0.9 |  |
| Osteopenia | Yes | SRT | 55 | N370S/N370S | NO | NO | NO | YES | NO | NO | NO | NO | -1.1 |  |
|  | Yes | SRT 5-10 | 54 | L444P/N370S | YES | NO | NO | YES | NO | NO | NO | NO | -1.4 |  |
|  | Yes | ERT >10 | 45 | N370S/N370S | NO | NO | NO | NO | NO | NO | YES | NO | -1.60 |  |
| Osteoporosis | Yes | ERT/SRT >10 | 47 | L444P/R493C | YES | YES | YES | YES | NO | NO | YES | NO | -2.0 |  |
|  | Yes | ERT 5-10 | 48 | N370S/N370S | NO | YES | YES | NO | YES | NO | NO | NO | -3.5 |  |
|  | Yes | SRT | 49 | N370S/N370S | NO | NO | YES | NO | YES | NO | NO | YES | -2.3 |  |
| **Age group >55** | | | | | | | | | | | | | | |
| Normal bone | Yes | ERT >10 | 59 | N370S/N370S | NO | NO | YES | YES | YES | NO | NO | YES |  | -0.5 |
| Osteopenia | Yes | ERT >10 | 64 | N370S/N370S | NO | NO | YES | YES | YES | NO | YES | YES |  | -1.6 |
|  | Yes | SRT 1-5 | 77 | N370S/N370S | YES | NO | YES | YES | YES | NO | NO | NO |  | -1.9 |
|  | Yes | ERT 1 year | 62 | N370S/D409H | YES | NO | YES | NO | NO | NO | NO | NO |  |  |
| Osteoporosis | Yes | SRT 5-10 | 60 | L444P/R502C | YES | NO | YES | YES | YES | NO | NO | NO |  | -2.4 |
|  | Yes | ERT >10 | 58 | L444P/N370S | NO | NO | YES | YES | YES | NO | NO | YES |  | -1.9 |
|  | Yes | SRT | 65 | N370S/N370S | NO | NO | YES | YES | YES | YES | NO | NO |  | -2.7 |
|  |  | naive | 66 | N370S | NO | NO | YES | YES | YES | NO | NO | NO |  | -2.7 |
|  |  | ERT 5-10 | 71 | N370S/R463C | YES | YES | YES | NO | YES | NO | NO | YES |  | -2.4 |

# Supplementary Tables 2

|  | **Controls** | | | **GD** | | |  | |  | |
| --- | --- | --- | --- | --- | --- | --- | --- | --- | --- | --- |
|  | **Average** | **STDEV** | **Average** | | **STDEV** | **T-test** | | **Fold Change** | |  |
| EGF | 65.15 | 9.11 | 146.48 | | 35.98 | **0.03** | | **2.25** | |  |
| Eotaxin | 31.50 | 6.32 | 33.47 | | 4.36 | 0.38 | | 1.06 | |  |
| FGF-2 | 79.12 | 13.15 | 155.82 | | 28.76 | **0.02** | | **1.97** | |  |
| FLT-3L | 14.44 | 4.38 | 13.12 | | 1.75 | 0.35 | | 0.91 | |  |
| Fractalkine | 197.96 | 57.35 | 231.35 | | 37.39 | 0.28 | | 1.17 | |  |
| G-CSF | 76.53 | 12.76 | 84.25 | | 15.44 | 0.35 | | 1.10 | |  |
| GM-CSF | 114.71 | 56.38 | 86.92 | | 25.76 | 0.29 | | 0.76 | |  |
| GROα | 35.66 | 7.24 | 64.56 | | 31.06 | 0.22 | | 1.81 | |  |
| IFNα2 | 86.39 | 13.60 | 136.25 | | 28.14 | 0.08 | | 1.58 | |  |
| IFNγ | 1.39 | 0.27 | 3.86 | | 0.91 | **0.01** | | **2.77** | |  |
| IL-1α | 60.75 | 17.53 | 93.59 | | 25.63 | 0.15 | | 1.54 | |  |
| IL-1β | 128.97 | 13.51 | 137.00 | | 22.40 | 0.39 | | 1.06 | |  |
| IL-1RA | 13.28 | 2.86 | 11.53 | | 3.02 | 0.33 | | 0.87 | |  |
| IL-2 | 2.70 | 0.44 | 6.50 | | 2.54 | 0.11 | | 2.41 | |  |
| IL-3 | 3.00 | 0.32 | 2.82 | | 0.47 | 0.38 | | 0.94 | |  |
| IL-4 | 2.42 | 0.39 | 4.73 | | 1.14 | **0.05** | | **1.95** | |  |
| IL-5 | 8.97 | 3.18 | 8.82 | | 3.26 | 0.49 | | 0.98 | |  |
| IL-6 | 3.79 | 0.76 | 3.16 | | 0.86 | 0.29 | | 0.84 | |  |
| IL-7 | 3.84 | 1.15 | 29.69 | | 25.05 | 0.19 | | 7.74 | |  |
| IL-8 | 27.63 | 16.40 | 7.63 | | 1.91 | 0.03 | | 0.28 | |  |
| IL-9 | 15.66 | 2.09 | 22.03 | | 3.11 | 0.05 | | 1.41 | |  |
| IL-10 | 5.74 | 2.86 | 5.44 | | 3.19 | 0.47 | | 0.95 | |  |
| IL-12p40 | 84.99 | 15.73 | 137.53 | | 40.19 | 0.14 | | 1.62 | |  |
| IL-12p70 | 4.72 | 0.69 | 20.88 | | 10.73 | 0.10 | | 4.42 | |  |
| IL-13 | 99.69 | 18.67 | 183.47 | | 34.00 | **0.03** | | **1.84** | |  |
| IL-15 | 9.13 | 1.33 | 16.00 | | 7.20 | 0.21 | | 1.75 | |  |
| IL-17A | 24.70 | 3.71 | 37.53 | | 13.90 | 0.22 | | 1.52 | |  |
| IL-17E/IL-25 | 5,517.83 | 603.43 | 7,350.03 | | 1,466.27 | 0.15 | | 1.33 | |  |
| IL-17F | 66.24 | 39.28 | 146.39 | | 66.50 | 0.17 | | 2.21 | |  |
| IL-18 | 190.30 | 64.67 | 82.94 | | 14.02 | 0.01 | | 0.44 | |  |
| IL-22 | 336.71 | 13.68 | 401.34 | | 24.58 | 0.02 | | 1.19 | |  |
| IL-27 | 990.97 | 158.60 | 1,495.13 | | 164.42 | 0.01 | | 1.51 | |  |
| IP-10 | 123.27 | 68.58 | 245.79 | | 127.22 | 0.22 | | 1.99 | |  |
| M-CSF | 163.60 | 47.00 | 72.51 | | 39.28 | 0.05 | | 0.44 | |  |
| MCP-1 | 202.07 | 27.11 | 240.96 | | 30.38 | 0.16 | | 1.19 | |  |
| MCP-3 | 21.16 | 1.32 | 34.52 | | 6.17 | **0.04** | | **1.63** | |  |
| MDC | 987.80 | 109.13 | 772.40 | | 84.43 | 0.04 | | 0.78 | |  |
| MIG/CXCL9 | 1,316.58 | 243.80 | 1,376.52 | | 431.27 | 0.45 | | 1.05 | |  |
| MIP-1α | 105.50 | 7.26 | 90.63 | | 9.73 | 0.12 | | 0.86 | |  |
| MIP-1β | 23.65 | 3.55 | 31.87 | | 8.25 | 0.21 | | 1.35 | |  |
| PDGF-AA | 620.26 | 154.60 | 1,186.84 | | 278.71 | **0.05** | | **1.91** | |  |
| RANTES | 4,664.78 | 620.06 | 2,602.31 | | 385.51 | 0.00 | | 0.56 | |  |
| sCD40L | 407.83 | 169.37 | 3,233.38 | | 1,019.67 | **0.01** | | **7.93** | |  |
| TGFα | 9.43 | 2.33 | 31.46 | | 14.08 | 0.10 | | 3.34 | |  |
| TNFα | 59.33 | 7.34 | 86.90 | | 19.81 | 0.13 | | 1.46 | |  |
| TNFβ | 82.45 | 7.11 | 93.21 | | 8.18 | 0.16 | | 1.13 | |  |
| VEGF-A | 75.08 | 22.29 | 214.18 | | 86.31 | 0.09 | | 2.85 | |  |
| 6CKine | 1,066.34 | 182.08 | 1,123.01 | | 131.98 | 0.38 | | 1.05 | |  |
| APRIL | 728.86 | 439.92 | 2,650.26 | | 723.37 | **0.02** | | **3.64** | |  |
| BAFF | 900.56 | 83.58 | 1,060.54 | | 133.65 | 0.17 | | 1.18 | |  |
| BCA-1 | 104.97 | 13.84 | 133.66 | | 15.66 | 0.08 | | 1.27 | |  |
| CCL28 | 166.13 | 16.78 | 342.17 | | 66.48 | **0.02** | | **2.06** | |  |
| CTACK | 614.70 | 73.07 | 1,002.98 | | 118.10 | **0.01** | | **1.63** | |  |
| CXCL16 | 998.40 | 108.81 | 864.28 | | 74.04 | 0.12 | | 0.87 | |  |
| ENA-78 | 1,107.51 | 235.30 | 1,213.10 | | 448.02 | 0.43 | | 1.10 | |  |
| Eotaxin-2 | 339.35 | 68.88 | 454.43 | | 166.85 | 0.28 | | 1.34 | |  |
| Eotaxin-3 | 106.25 | 11.03 | 133.08 | | 13.65 | 0.07 | | 1.25 | |  |
| GCP-2 | 78.93 | 24.73 | 212.29 | | 70.54 | 0.06 | | 2.69 | |  |
| Granzyme A | 51.40 | 14.93 | 103.12 | | 20.08 | **0.02** | | **2.01** | |  |
| Granzyme B | 9.21 | 2.10 | 39.34 | | 17.73 | 0.08 | | 4.27 | |  |
| HMGB1 | 5,296.69 | 311.83 | 5,877.69 | | 472.03 | 0.16 | | 1.11 | |  |
| I-309 | 8.81 | 2.07 | 32.32 | | 25.56 | 0.22 | | 3.67 | |  |
| I-TAC | 115.57 | 27.28 | 208.45 | | 52.63 | 0.08 | | 1.80 | |  |
| IFNβ | 92.80 | 12.11 | 130.14 | | 15.32 | 0.03 | | 1.40 | |  |
| IFNω | 26.34 | 4.52 | 45.63 | | 12.93 | 0.11 | | 1.73 | |  |
| IL-11 | 40.79 | 2.89 | 48.61 | | 4.02 | 0.06 | | 1.19 | |  |
| IL-16 | 567.63 | 168.50 | 583.24 | | 184.95 | 0.47 | | 1.03 | |  |
| IL-20 | 78.17 | 11.31 | 97.04 | | 20.36 | 0.23 | | 1.24 | |  |
| IL-21 | 15.15 | 1.40 | 28.92 | | 6.96 | **0.05** | | **1.91** | |  |
| IL-23 | 3,087.67 | 1,274.19 | 10,361.29 | | 4,580.75 | 0.19 | | 3.36 | |  |
| IL-24 | 1,014.33 | 103.09 | 1,555.02 | | 167.89 | **0.01** | | **1.53** | |  |
| IL-28A | 55.14 | 4.82 | 68.27 | | 5.95 | 0.05 | | 1.24 | |  |
| IL-29 | 36.01 | 6.38 | 40.09 | | 7.51 | 0.34 | | 1.11 | |  |
| IL-31 | 25.14 | 7.75 | 68.78 | | 25.50 | 0.08 | | 2.74 | |  |
| IL-33 | 29.89 | 5.48 | 82.99 | | 37.55 | 0.12 | | 2.78 | |  |
| IL-34 | 127.22 | 14.67 | 158.69 | | 19.75 | 0.11 | | 1.25 | |  |
| IL-35 | 153.94 | 49.88 | 505.52 | | 168.25 | **0.04** | | **3.28** | |  |
| LIF | 20.41 | 6.74 | 25.14 | | 7.32 | 0.31 | | 1.23 | |  |
| Lymphotactin | 71.74 | 5.81 | 81.36 | | 6.35 | 0.13 | | 1.13 | |  |
| MCP-2 | 31.80 | 9.54 | 36.12 | | 8.11 | 0.35 | | 1.14 | |  |
| MCP-4 | 61.33 | 9.17 | 93.74 | | 14.07 | **0.04** | | **1.53** | |  |
| MIP 1δ | 7,799.61 | 1,773.17 | 7,595.30 | | 2,377.85 | 0.48 | | 0.97 | |  |
| MIP-3α | 25.32 | 12.84 | 16.86 | | 2.64 | 0.16 | | 0.67 | |  |
| MIP-3β | 57.37 | 15.36 | 117.15 | | 21.42 | **0.02** | | **2.04** | |  |
| MPIF-1 | 274.62 | 60.21 | 416.54 | | 56.34 | **0.03** | | **1.52** | |  |
| Perforin | 6,992.26 | 787.96 | 6,188.79 | | 679.17 | 0.20 | | 0.89 | |  |
| sCD137 | 14.87 | 2.66 | 21.14 | | 3.58 | 0.09 | | 1.42 | |  |
| SCF | 1,110.05 | 113.05 | 901.78 | | 99.83 | 0.07 | | 0.81 | |  |
| SDF-1 | 3,488.80 | 518.22 | 4,147.06 | | 400.92 | 0.13 | | 1.19 | |  |
| sFas | 21.85 | 1.98 | 18.74 | | 2.04 | 0.13 | | 0.86 | |  |
| sFasL | 174.94 | 22.95 | 488.75 | | 437.52 | 0.27 | | 2.79 | |  |
| TARC | 57.89 | 14.88 | 72.15 | | 16.38 | 0.25 | | 1.25 | |  |
| TPO | 223.95 | 60.46 | 548.70 | | 125.92 | **0.02** | | **2.45** | |  |
| TRAIL | 41.12 | 6.56 | 59.01 | | 8.52 | 0.05 | | 1.44 | |  |
| TSLP | 1.73 | 0.15 | 2.41 | | 0.24 | 0.01 | | 1.39 | |  |

# Supplementary Tables 3

Correlation analysis was conducted to evaluate the relationships between the Z-scores of bone mineral density (BMD), age, and serum levels of the cytokines Eotaxin (CCL11), MCP-1 (CCL2), MIP-3β (CCL19), and soluble STACK (TNFSF13B). This analysis found no significant differences in cytokine levels and BMD score among female patients with GD under 55 years old. Pearson correlation analysis was performed with a 95% confidence interval and a one-tailed approach.

|  | Z score  vs. Eotaxin | Z score  vs. MCP-1 | Z score  vs. MIP-3β | Z score  vs. CTACK | Z score  vs. Age |
| --- | --- | --- | --- | --- | --- |
| Pearson r |  |  |  |  |  |
| r | -0.2738 | -0.09739 | 0.07255 | -0.1054 | 0.04658 |
| 95% confidence interval | -0.6775 to 0.2568 | -0.5658 to 0.4185 | -0.4389 to 0.5485 | -0.5713 to 0.4118 | -0.4597 to 0.5300 |
| R squared | 0.07496 | 0.009486 | 0.005264 | 0.01112 | 0.002170 |
| P (one-tailed) | 0.1524 | 0.3599 | 0.3947 | 0.3488 | 0.4320 |
| P value summary | ns | ns | ns | ns | ns |
| Significant? (alpha = 0.05) | No | No | No | No | No |
| Number of XY Pairs | 16 | 16 | 16 | 16 | 16 |

# Supplementary Tables 4

Correlation analysis was conducted to evaluate the relationships between the T-scores of BMD, age, and serum levels of the cytokines Eotaxin (CCL11), MCP-1 (CCL2), MIP-3β (CCL19), and soluble STACK (TNFSF13B). This analysis found no significant differences in cytokine levels and BMD scores among female patients with GD aged 55 years and older. Pearson correlation analysis was performed with a 95% confidence interval and a one-tailed approach.

|  | T score  vs. Eotaxin | T score  vs. MCP-1 | T score  vs. MIP-3β | T score  vs. CTACK | T score  vs. Age |
| --- | --- | --- | --- | --- | --- |
| Pearson r |  |  |  |  |  |
| r | 0.2420 | -0.2778 | -0.6784 | -0.6326 | 0.01463 |
| 95% confidence interval | -0.7088 to 0.8806 | -0.8889 to 0.6891 | -0.9609 to 0.2962 | -0.9542 to 0.3678 | -0.8065 to 0.8165 |
| R squared | 0.05857 | 0.07717 | 0.4603 | 0.4001 | 0.0002141 |
| P (one-tailed) | 0.3220 | 0.2970 | 0.0692 | 0.0889 | 0.4890 |
| P value summary | ns | ns | ns | ns | ns |
| Significant? (alpha = 0.05) | No | No | No | No | No |
| Number of XY Pairs | 6 | 6 | 6 | 6 | 6 |

## Supplementary Figures

**Supplementary Figure 1.** Supplemental Figure 1. Circulated levels of MCP-1, MCP4, and TNF-α. (A) The levels of MCP-1 in healthy control subjects (Control) and patients with GD. (B) The levels of MCP-1 in Control and in patients with GD categorized by bone status: no bone complications (N), osteopenia (OSN), and osteoporosis (OSR).who have no bone complications (N), osteopenia (OSN), and osteoporosis (OSR). Statistical significance was determined with *P < 0.05, two-tailed T-tests. (C) The correlation between age and the circulating MCP-4 level in healthy controls. The MCP-4 level was measured using ELISA. (D) The levels of TNF-α in healthy control subjects and patients with GD. The TNF-α level was measured using ELISA. Statistical significance was determined with *P < 0.05, two-tailed T-tests. (E) TNF-α levels in female patients with GD categorized by bone status: no bone complications (N), osteopenia (OSN), and osteoporosis (OSR).
